# Performance of Comprehensive Complication Index and Clavien-Dindo Complication Scoring System in Liver Surgery for Hepatocellular Carcinoma

**DOI:** 10.3390/cancers12123868

**Published:** 2020-12-21

**Authors:** Alessandro Giani, Federica Cipriani, Simone Famularo, Matteo Donadon, Davide Paolo Bernasconi, Francesco Ardito, Federico Fazio, Daniele Nicolini, Pasquale Perri, Mario Giuffrida, Nicholas Pontarolo, Matteo Zanello, Quirino Lai, Simone Conci, Sarah Molfino, Paola Germani, Enrico Pinotti, Maurizio Romano, Giuliano La Barba, Cecilia Ferrari, Stefan Patauner, Alberto Manzoni, Ivano Sciannamea, Luca Fumagalli, Albert Troci, Valentina Ferraro, Antonio Floridi, Fabrizio Romano, Cristina Ciulli, Marco Braga, Francesca Ratti, Guido Costa, Francesco Razionale, Nadia Russolillo, Laura Marinelli, Valerio De Peppo, Elena Cremaschi, Francesco Calabrese, Zoe Larghi Laureiro, Giovanni Lazzari, Davide Cosola, Mauro Montuori, Luca Salvador, Alessandro Cucchetti, Angelo Franceschi, Michele Ciola, Valentina Sega, Pietro Calcagno, Luca Pennacchi, Michele Tedeschi, Riccardo Memeo, Michele Crespi, Marco Chiarelli, Adelmo Antonucci, Giuseppe Zimmitti, Antonio Frena, Andrea Percivale, Giorgio Ercolani, Giacomo Zanus, Mauro Zago, Paola Tarchi, Gian Luca Baiocchi, Andrea Ruzzenente, Massimo Rossi, Elio Jovine, Marcello Maestri, Raffaele Dalla Valle, Gian Luca Grazi, Marco Vivarelli, Alessandro Ferrero, Felice Giuliante, Guido Torzilli, Luca Aldrighetti, Luca Gianotti

**Affiliations:** 1 School of Medicine and Surgery, University of Milano-Bicocca, 20900 Monza, Italy; a.giani3@gmail.com (A.G.); simone.famularo@gmail.com (S.F.); fabrizio.romano@unimib.it (F.R.); c.ciulli@campus.unimib.it (C.C.); marco.braga@unimib.it (M.B.); 2 Hepatobiliary Surgery Division, Ospedale San Raffaele, 20132 Milan, Italy; cipriani.federica@hsr.it (F.C.); ratti.francesca@hsr.it (F.R.); aldrighetti.luca@hsr.it (L.A.); 3 Department of Hepatobiliary and General Surgery, Humanitas University, Humanitas Clinical and Research Center, Rozzano, 20089 Milan, Italy; matteo.donadon@hunimed.eu (M.D.); guido.costa@humanitas.it (G.C.); guido.torzilli@humanitas.it (G.T.); 4 Bicocca Bioinformatics Biostatistics and Bioimaging Centre-B4, School of Medicine and Surgery, University of Milano-Bicocca, 20900 Monza, Italy; davide.bernasconi@unimib.it; 5 Hepatobiliary Surgery Unit, Fondazione Policlinico Universitario A. Gemelli, IRCCS, Catholic University of the Sacred Heart, 00118 Rome, Italy; francesco.ardito@unicatt.it (F.A.); francescorazionale@gmail.com (F.R.); felice.giuliante@unicatt.it (F.G.); 6 Department of General and Oncological Surgery, Mauriziano Hospital “Umberto I”, 10112 Turin, Italy; ffazio@mauriziano.it (F.F.); nrussolillo@mauriziano.it (N.R.); aferrero@mauriziano.it (A.F.); 7 Hepatopancreatobiliary and Transplant Unit, Department of Experimental and Clinical Medicine, Polytechnic University of Marche, 60121 Ancona, Italy; daniele.nicolini@ospedaliriuniti.marche.it (D.N.); dr.lauramarinelli@gmail.com (L.M.); vivarelli63@libero.it (M.V.); 8 Division of Hepatobiliarypancreatic surgery, IRCCS—Regina Elena National Cancer Institute, 00119 Rome, Italy; perri.pasquale@gmail.com (P.P.); valerio.depeppo2794@gmail.com (V.D.P.); gianluca.grazi@ifo.gov.it (G.L.G.); 9 Department of Medicine and Surgery, University of Parma, 43126 Parma, Italy; mario.giuffrida4@gmail.com (M.G.); elena.cremaschi@yahoo.com (E.C.); raffaele.dallavalle@unipr.it (R.D.V.); 10 Unit of General Surgery 1, University of Pavia and Foundation IRCCS Policlinico San Matteo, 27100 Pavia, Italy; nicholas.pontarolo@gmail.com (N.P.); francesco.calabrese01@universitadipavia.it (F.C.); marcello.maestri@unipv.it (M.M.); 11 Department of Surgery, AOU Sant’Orsola Malpighi, IRCCS Azienda Ospedaliera Universitaria, 40141 Bologna, Italy; m.zanello@ausl.bologna.it (M.Z.); elio.jovine@ausl.bologna.it (E.J.); 12 Hepatobiliary and Organ Transplantation Unit, Sapienza University of Rome, Umberto I Polyclinic of Rome, 00118 Rome, Italy; lai.quirino@libero.it (Q.L.); zll_89@hotmail.it (Z.L.L.); massimo.rossi@uniroma1.it (M.R.); 13 Division of General and Hepatobiliary Surgery, Department of Surgical Sciences, Dentistry, Gynecology and Pediatrics, University of Verona, 37121 Verona, Italy; simoneconci83@gmail.com (S.C.); giovanni.lazzari@studenti.univr.it (G.L.); ruzzenentea@gmail.com (A.R.); 14 Department of Clinical and Experimental Sciences, University of Brescia, 25136 Brescia, Italy; sarahmolfino@gmail.com (S.M.); gianluca.baiocchi@unibs.it (G.L.B.); 15 Department of General Surgery, ASUGI, University Hospital of Trieste, 34121 Trieste, Italy; paolagermani1987@gmail.com (P.G.); dcosola84@gmail.com (D.C.); paolatarchi@hotmail.com (P.T.); 16 Department of Surgery, Ponte San Pietro Hospital, 24129 Bergamo, Italy; enricopinotti@hotmail.it (E.P.); mauro.montuori@hotmail.it (M.M.); maurozago.md@gmail.com (M.Z.); 17 Department of Surgical, Oncological and Gastroenterological Science (DISCOG), University of Padua; Hepatobiliary and Pancreatic Surgery Unit—Treviso Hospital, 31102 Treviso Italy; maurizio.romano@aulss2.veneto.it (M.R.); lucasalvador91@yahoo.it (L.S.); giacomo.zanus@aulss2.veneto.it (G.Z.); 18 General and Oncologic Surgery, Morgagni-Pierantoni Hospital, 47122 Forlì, Italy; labarba.giuliano@gmail.com (G.L.B.); aleqko@libero.it (A.C.); giorgio.ercolani@auslromagna.it (G.E.); 19 HPB Surgical Unit, San Paolo Hospital, 17100 Savona, Italy; cecia8981@gmail.com (C.F.); a.franceschi@asl2.liguria.it (A.F.); a.percivale@gmail.com (A.P.); 20 Department of Surgery, Bolzano Central Hospital, 39100 Bolzano, Italy; stefan.patauner@sabes.it (S.P.); michele.ciola@sabes.it (M.C.); antonio.frena@sabes.it (A.F.); 21 Department of General Surgery, Poliambulanza Foundation Hospital, 25136 Brescia, Italy; alberto.manzoni@poliambulanza.it (A.M.); valentina.sega@poliambulanza.it (V.S.); giuseppe.zimmitti@poliambulanza.it (G.Z.); 22 Department of Surgery, Monza Polyclinic, 20900 Monza, Italy; ivano.sciannamea@gmail.com (I.S.); adelmo.antonucci@policlinicodimonza.it (A.A.); 23 Department of Emergency and Robotic Surgery, ASST Lecco, 23900 Lecco, Italy; lu.fumagalli@asst-lecco.it (L.F.); pietro.calcagno89@gmail.com (P.C.); mee.chiarelli@gmail.com (M.C.); 24 Department of Surgery, L. Sacco Hospital, 20143 Milan, Italy; troci.albert@asst-fbf-sacco.it (A.T.); pennacchi.luca@asst-fbf-sacco.it (L.P.); crespi.michele@asst-fbf-sacco.it (M.C.); 25 Department of Hepato-Pancreatic-Biliary Surgery, Miulli Hospital, 70132 Bari, Italy; ferrarov.v@libero.it (V.F.); mictedeschi@yahoo.it (M.T.); info@drmemeoriccardo.com (R.M.); 26 Department of General Surgery, ASST Crema, 26013 Crema, Italy; antoniofloridi@hotmail.com; 27 HPB Unit, Department of Surgery, San Gerardo Hospital, 20900 Monza, Italy

**Keywords:** liver surgery, hepatocellular carcinoma, Clavien-Dindo classification, comprehensive complication index, performance, length of stay, morbidity

## Abstract

**Simple Summary:**

The comprehensive complication index (CCI) and the Clavien-Dindo Complication (CDC) scoring system are two metrics designed to quantify the burden of postoperative morbidity. We performed a retrospective study retrieving data from a multi-institutional Italian register. The aim was to compare the performance of the two metrics in predicting excessive length of hospital stay (e-LOS) of patients who underwent liver resections for hepatocellular carcinoma. A total of 2669 patients were analyzed. A derivation (*n* = 1345) and validation sets (*n* = 1324) were created to test the strength of results. In both cohorts, the analysis showed that CCI was slightly superior in predicting e-LOS in complicated patients. The accuracy of CCI was even better when considering a subgroup of patients who experienced at least two complications. The results of this population-specific analysis suggest that CCI is preferable in weighting postoperative morbidity burden.

**Abstract:**

Background: We aimed to assess the ability of comprehensive complication index (CCI) and Clavien-Dindo complication (CDC) scale to predict excessive length of hospital stay (e-LOS) in patients undergoing liver resection for hepatocellular carcinoma. Methods: Patients were identified from an Italian multi-institutional database and randomly selected to be included in either a derivation or validation set. Multivariate logistic regression models and ROC curve analysis including either CCI or CDC as predictors of e-LOS were fitted to compare predictive performance. E-LOS was defined as a LOS longer than the 75th percentile among patients with at least one complication. Results: A total of 2669 patients were analyzed (1345 for derivation and 1324 for validation). The odds ratio (OR) was 5.590 (95%CI 4.201; 7.438) for CCI and 5.507 (4.152; 7.304) for CDC. The AUC was 0.964 for CCI and 0.893 for CDC in the derivation set and 0.962 vs. 0.890 in the validation set, respectively. In patients with at least two complications, the OR was 2.793 (1.896; 4.115) for CCI and 2.439 (1.666; 3.570) for CDC with an AUC of 0.850 and 0.673, respectively in the derivation cohort. The AUC was 0.806 for CCI and 0.658 for CDC in the validation set. Conclusions: When reporting postoperative morbidity in liver surgery, CCI is a preferable scale.

## 1. Introduction

Hepatic resection offers the best chance of long-term survival for patients with resectable hepatocellular carcinoma (HCC) [1,2]. Albeit perioperative mortality following liver surgery decreased over the past decades to less than 5%, morbidity still occurs frequently in a range of 20–40% depending mainly on the extent of resection, the underlying patient liver function, and the reporting scales [3,4,5]. Therefore, it might be of value to identify objective and reproducible metrics for scaling the magnitude of complications, to achieve quality control, and to compare outcomes among institutions.

The Clavien-Dindo classification (CDC), originally described in 2004 [6], is the most broadly grading system used for weighting postoperative morbidity (more than 14,000 citations by November 2020. Scopus.com). Even if the CDC is an objective, simple, and reproducible classification, it carries the limitation of scaling the entire postoperative course by the single most serious complication occurred. To overcome this disadvantage, in 2013 the same institution proposed a new scale, the comprehensive complication index (CCI) [7], that incorporates all complications and their severity as defined by the CDC and summarizes postoperative morbidity with a numerical scale ranging from 0 to 100.

Despite that CCI and CDC scoring systems are closely related metrics, CCI allows a longitudinal assessment of morbidity because the addition of a complication, appearing at a later time-point, is added to the score. By this computation CCI appears more precise to capture the overall morbidity burden [8,9]. However, comparison of the two scoring systems have been mostly applied in studies with substantial case-mix and heterogeneous populations [10,11,12]. As it may be more desirable to analyze populations with defined intervention-specific complications, we aimed to assess the performance of CCI and CDC in predicting LOS and excessive LOS (e-LOS) in patients undergoing liver resection for HCC. Length of hospital stay (LOS) can be considered as a reliable proxy of surgical morbidity, since complicated clinical courses generally result in a longer duration of hospitalization [13].

## 2. Material and Methods

### 2.1. Study Overview and Population

Patients who underwent liver resection for HCC with curative intent between 2007 and 2018 were identified from an Italian multi-institutional database. Patient data were retrieved retrospectively from this dataset, promoted by the Hepatocarcinoma Recurrence in the Liver Study (He.Rc.O.Le.S.) group. The study protocol followed the ethical guidelines of the 1975 Declaration of Helsinki (as revised in Brazil 2013). The Ethical Committee of the coordinating center (University of Milano-Bicocca; San Gerardo Hospital, Monza) reviewed and approved the protocol (211218HSG-R) on 21 December 2018. Inclusion criteria were: (1) first diagnosis of HCC without any previous treatment; (2) age ≥ 18 years; (3) HCC diagnosis confirmed at histology. Exclusion criteria were: (1) surgery as a down-staging therapy for transplant; (2) patients who eventually underwent liver transplantation; (3) mixed primary liver cancers (e.g., hepatocholangiocarcinoma). All data were anonymized prior to submission to the coordinating center. Data collection was performed using the same electronic database at all centers.

Centers were randomly selected to be included in either the derivation or validation set to obtain a similar number of patients in the two cohorts.

Results are reported according to principles of Strengthening the Reporting of Observational Studies in Epidemiology (STROBE) [14].

### 2.2. Variables and Definitions

Age, sex, and liver function were recorded at the first office visit. The presence of cirrhosis and its severity (Child-Pugh score) was evaluated and graded by expert hepatologists. Presence of HCV or HBV infection and serum biochemical values of bilirubin, albumin, platelets, and international normalized ratio (INR) were also recorded at baseline. The American Society of Anesthesiologists (ASA) class was assessed during the preoperative patient evaluation. The number and diameter of nodules were assessed through preoperative radiologic imaging and confirmed by intraoperative ultrasound. The extension resection was defined as minor when ≤3 liver segments were resected and major when >3 segments, according to the Brisbane nomenclature [15]. The definitions of anatomic resection (AR) and parenchyma-sparing resection (PSR) were previously reported [16]. Other surgery-related variables were minimally invasive approach, intraoperative blood transfusion, and duration of operation. LOS was calculated from the day of operation to hospital discharge. e-LOS was defined as the LOS longer than the 75th percentile among patients who experienced at least one complication. Post-operative complications grading was recorded according to both CDC [6] and CCI [7]. Post-operative mortality was calculated as the number of deaths occurring within 90 days from surgery; these patients were excluded from the calculation of LOS and e-LOS. Center volume was stratified according to the number of liver resections performed per year: ≤50 procedures identified a low-volume center, 51–100 resections a medium-volume center, and >100 procedures a high-volume center [17].

### 2.3. Endpoints

The primary endpoint was to assess the performance of CCI and CDC to predict LOS and e-LOS in patients undergoing liver resection for HCC.

The secondary endpoint was to find an optimal CCI cut-off value capable of predicting e-LOS.

### 2.4. Statistical Analysis

Sample description was performed using median and interquartile range (IQR) for numeric variables and number and proportion for categorical variables, for both derivation and validation sets.

The distribution of CCI in each CDC category was explored graphically using boxplots, while the association between each score and LOS was represented with a scatter plot, again in both datasets.

After excluding patients who died within 90 days (14 in the derivation set and 20 in the validation set), the association of each score with LOS (log-transformed) was analyzed using linear regression. We fit univariate models, including CCI or CDC as the only covariate, and multivariate models, adjusted by type of surgery (major vs. minor), open vs. laparoscopy, age (per year), ASA score (1–2 vs. 3–4), Child grade (A vs. B), duration of surgery (>4 h vs. ≤4 h), center volume (high vs. medium/low) on the derivation set. We evaluated and compared the goodness-of-fit of the models including CCI or CDC using the R-squared and the root mean squared error (RMSE) indexes. This was done on the derivation set and, on the validation set without refitting the models. The whole linear regression analysis was repeated considering, in both sets, only the subgroup of patients with at least two postoperative complications.

The association of both scores with e-LOS was analyzed using logistic regression. Again, we fit univariate and multivariate models (adjusting for the same covariates used for the analysis of LOS) on the derivation set excluding patients who died during hospital stay. The discriminatory ability of the models to identify patients with e-LOS was evaluated using the area under the ROC curve (AUC) index both on the derivation and validation (without refitting the models) sets. Again, the whole logistic regression analysis was repeated considering, in both sets, only the subgroup of patients with at least two postoperative complications.

Finally, considering only the subset of patients with at least one postoperative complication in both sets, we built an ROC curve to find the optimal cut-off (corresponding to the maximum Youden index) of CCI to be used in order to identify patients with e-LOS. We repeated the analysis within strata defined by minor/major surgery, open/laparoscopic surgery, presence/absence of cirrhosis and Child grade A/B. The R software version 4.0.1 was used for all the analyses.

## 3. Results

### 3.1. Descriptive Findings

The analysis was carried out in May 2020, and at that time the records of 2917 patients, collected in the 25 centers, were entered into the register. A total of 248 records were excluded because of missing information on LOS, CDC or CCI, resulting in 2669 patients available for the final analysis. The final sample size of the derivation and validation cohorts were 1345 and 1324 records, respectively.

The main characteristics of the two cohorts are summarized in Table 1. Regarding the derivation set, the median age was 70.0 years (IQR: 63.0–75.0), 328 patients were female (24.4%) and ASA score ≥ 3 was assigned to 611 (46.7%). Major surgery was performed in 293 patients (21.8%), laparoscopic resection in 568 (42.2%), and 1175 (90.9%) had a well-preserved underlying liver function (Child A). The median duration of the operation was 230 min (IQR: 170.0–290.0) and 118 cases (9.1%) needed intraoperative blood transfusions. The overall complication rate was 33.5% (*n* = 451), and 7.0% (*n* = 95) had a CDC ≥ 3. The overall median CCI was 0 (IQR: 0–20.9) and the mean 8.6 (±16.0 SD), while considering only patients with at least one complication the median CCI was 20.9 (IQR 20.9–26.6) and the mean 25.5 (±18.1 SD). The overall median LOS was 7.0 days (5.0–9.0 IQR), 10.0 days (IQR 8.0–15.0) among complicated patients, and e-LOS was thus considered as LOS ≥ 15 days. A total of 260 patients (19.3%) had only 1 complication, and 191 (14.2%) had at least 2 complications. The 90-day mortality was 1% (14 cases). Patients included in the validation set presented similar complication rate (40.4%), multiple complication rate (13.1%), median CCI (20.9, IQR: 8.7–29.60), and median LOS (9, IQR: 7–12). In this cohort, patients had a better underlying liver function (97% of Child-Pugh Grade A), lower rate of cirrhosis (56.1%), fewer underwent major surgery (20.1%), and the majority was operated in high volume centers (77.9%).

**Figure 1:**
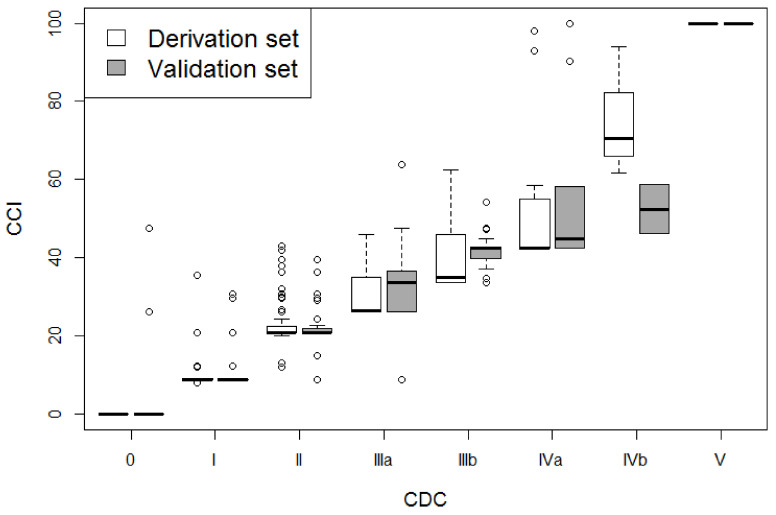
Boxplot showing the association between comprehensive complication index (CCI) and Clavien-Dindo classification (CDC) scores in the derivation and validation sets.

Figure 1 shows the distribution of CCI in categories of CDC for both derivation and validation sets. The Spearman index was high indicating a strong correlation between the two scores (99.4% overall and 87.6% in complicated patients in the derivation set; 99.0% overall and 92.9% in complicated patients in the validation set).

### 3.2. Association of CCI and CDC with Postoperative LOS and e-LOS

**Figure 2:**
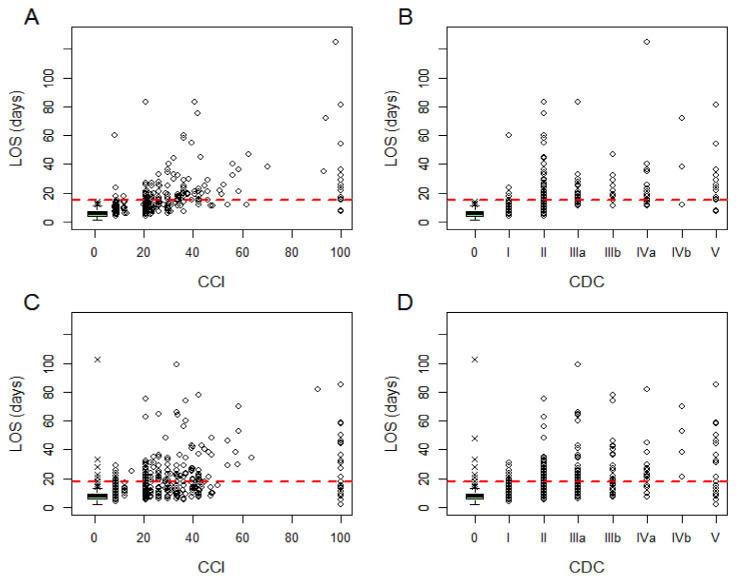
Scatter plots of the distribution of length of hospital stay (LOS). (**A**) Comprehensive complication index (CCI) in the derivation set. (**B**) Clavien-Dindo classification (CDC) in the derivation set; (**C**) comprehensive complication index (CCI) in the validation set; (**D**) Clavien-Dindo classification (CDC) in the validation set. The distribution of LOS for patients with CCI and CDC = 0 is represented with a boxplot rather than with raw dots. The dashed red line represents the e-LOS = 15 days (75th percentile of LOS among patients with at least one complication in the derivation set). Points that lie above the horizontal red line represent patients with e-LOS.

The ability of CDC and CCI to measure postoperative morbidity was evaluated in terms of association with postoperative LOS and e-LOS. Figure 2A–D show the relationship between LOS and the two scoring systems in derivation and validation sets.

The multivariate linear regression analysis on the derivation set showed that the expected mean log (LOS) change, per 10 units of CCI increment, was 0.27 (95%CI: 0.25–0.28), corresponding to an average 31% increment of LOS, while for category increment of CDC it was 0.29 (95%CI: 0.27–0.31), corresponding to an average 34% increment of LOS. The goodness of fit of the CCI model was slightly superior to the CDC model as indicated by the higher R^2^ and lower RMSE, also in the validation set. Larger differences between the performance of CCI vs. CDC models were observed in the subset of patients with at least two complications (Table 2).

The coefficients of all covariates included in the linear models are shown in Supplementary Materials Table S1.

At the multivariate logistic regression for e-LOS in the derivation set, the odds ratio for 10 units of CCI increment, was 5.60 (95%CI: 4.20–7.44) vs. 5.51 (95%CI: 4.15–7.30) for CDC category increment with a moderately higher discriminatory ability for the CCI model even in the validation set (AUC = 0.893 for CCI vs. 0.890 for CDC). CCI showed an even higher ability to discriminate patients with e-LOS than CDC in the subset of patients with at least two complications (Table 3). The odds ratios of all covariates included in the logistic models are shown in Table S2.

**Figure 3:**
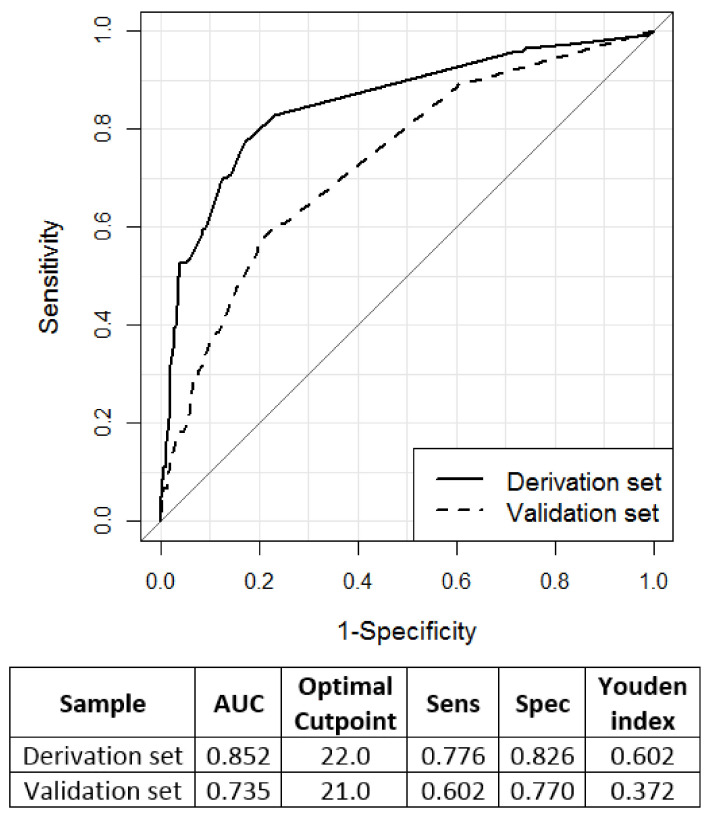
ROC curve showing the performance of CCI in identifying patients with e-LOS in the derivation and validation sets. The AUC values and the optimal cut-point identified by the Youden Index with the corresponding sensitivity and specificity are also reported.

The performance of CCI, evaluated by the ROC curve methodology, to identify—amongst patients with at least one complication—those with e-LOS is reported in Figure 3 (derivation and validation sets). Considering a CCI score of 22 (optimal cut-point at the Youden index) as a predictor of e-LOS, the sensitivity was 77.6% and the specificity 82.6% in the derivation set. The AUC index was 0.852, decreasing to 0.735 when the validation set was considered indicating, even so, a good discriminatory performance of CCI.

This was confirmed even when subgroups were analyzed, although slightly different cut-points were found (Figure S1A–H).

## 4. Discussion

In grading a complicated postoperative course, the CDC scoring system [6] accounts for the most severe adverse event and this may underestimate a more encompassing representation of surgery-related morbidity. Failure to capture the number and severity of every single complication may result in a partial report of the characteristics of a postoperative course. The CCI scale has been created to outline more accurately the overall morbidity burden since it integrates in one formula all documented complications weighted by severity [7]. The values of the CCI range in a numeric scale from 0 up to 100 and thus this metric theoretically grants a wider and more differentiated grading of complications than CDC.

A comparison of the two scoring systems has been already applied in several studies investigating broad case-mix and heterogeneous populations [8,10,11,18,19,20]. However, different types of surgical procedures expose patients to peculiar complications and different risks according to the technical details, magnitude of injury, and baseline characteristics of the population. As it may be more accurate to analyze intervention-specific complications, we aimed to assess and compare the ability of CCI and CDC to predict LOS in a more homogeneous cohort, i.e., patients undergoing liver resection for HCC. This is a worthy subset of patients to be analyzed because of the probability of encountering multiple postoperative complications, for both the complexity of surgery and the patient-inherent risks, mostly related to the underlying liver function [21,22,23].

The present study advocates that both CCI and CDC scoring systems perform well in predicting the duration of hospitalization after liver resection for HCC. However, CCI was slightly superior to CDC in predicting both overall LOS and e-LOS in complicated patients, in both the derivation cohort and in the validation one. In the validation set there were less cirrhotic patients, less CDC 0, longer LOS, less laparoscopic cases, and less anatomical resections but more high-volume centers. Center volume has been repeatedly described as a variable affecting morbidity. Since we conducted a multi-institutional study, we randomly assigned centers to the validation or the derivation cohort to limit this bias. However, the two cohorts were partially not homogeneous. Hence, all multivariate models were adjusted for several confounders including center volume.

Although the overall difference within metrics performance was marginal in the overall sample, the association between CCI and LOS held stronger than CDC especially in the subset of patients with at least two complications. This suggests that CCI better captured any event affecting longer hospitalization. These statements are supported by the results of the linear regression analysis showing that the CCI models were always slightly better than the CDC models. Similarly, the multivariate logistic regression analysis suggested that the areas under the ROC curves of the CCI models were always greater than the CDC models suggesting that CCI had a better ability to discriminate patients with longer LOS. Therefore, the overall findings imply that CCI is a more accurate system in grading the morbidity burden, eventually affecting the duration of hospitalization.

It can be argued that our results indicate a marginal advantage of CCI over CDC in predicting LOS and e-LOS in complicated patients. This can be partially explained by the low proportion of patients who experienced at least two complications (14.2%). Accordingly, when more than 85% of the population has no or only one complication, the two scores overlap by definition and so the comparison is futile [7]. For this reason, the accuracy of CCI is only marginally better when the overall cohort is considered, but this grading system, as expected, becomes more accurate in describing patients with multiple complications.

Despite the slightly better performance of CCI, this metric has some hindrances in predicting e-LOS. In fact, CCI was not evenly distributed through the scale and the dissemination tends to cluster in values embodying each grade of CDC for patients facing only one adverse event. Moreover, in patients with a postoperative course characterized by more severe complications (CDC > II), a wider spectrum of CCI values was present. This might be because severe complications are often coupled with additional minor ones. Otherwise, low-grade complications may not necessarily prolong hospitalization. Similar uneven CCI distribution has been described by Kim et al. [24] in a series of patients who underwent gastric resection for cancer.

The ROC curve analysis of CCI for complicated patients with e-LOS found a score of 22 as the optimal cut-point for defining patients who experienced a delayed hospital discharge. Thus, this CCI cut-off value can be used to dichotomize the study population into low- and high-risk of having e-LOS. A CCI score greater than 22, would mean that a patient had at least two minor complications, according to CDC (notably, at least one complication graded I and another graded II), or a major one (CDC ≥ III). The CDC grade IIIA—which often defines “major” or “severe” morbidity [6]—corresponds to a calculated CCI value of 26.2 [7]. The cut-off value of CCI obtained in our series was slightly lower. It is possible that in the context of liver resection for HCC, e-LOS might be more affected by the occurrence of multiple minor adverse events and a lower CCI score better defines a complicated postoperative course. The data of the derivation set were tested against a validation cohort. The results suggested a good performance of the CCI adding strength to the reproducibility and accuracy of the findings. However, future prospective studies are warranted to confirm this CCI value as a reliable threshold for defining excessive LOS after liver resection. External validation is appropriate because clinical management, discharge criteria, patient-related variables, and health or social care organization may substantially differ in other settings.

Few authors compared the performance of CCI and CDC in other homogeneous surgical procedures, namely, gastrostomies, intestinal resections, cystectomies, liver transplantations, esophagectomies and pancreatectomies [20,24,25,26,27,28,29,30]. Their results are quite consistent with ours in defining CCI as a more precise scale for reporting postoperative morbidity. Reporting CCI in surgical literature may have additional value, considering its capability of monitoring outcomes for individual surgeons [25] or for investigating historical trends of departments in terms of perioperative results after hepatectomies [31].

The retrospective study design implies several drawbacks. First, the duration of hospital stay in absence of a priori definition of discharge criteria, may be affected by social and logistic factors, and by the confidence of a single patient to go home safely. However, since there is no gold standard for measuring clinical outcomes and comparing clinical implications of different complication grades, we considered LOS as a surrogate marker for surgical outcome, as in other studies [8,20,26,27]. Second, medical costs may also be a good endpoint to reflect the burden of postoperative course [9,18], but our dataset was not designed to collect economic parameters. Third, readmission rate or new admission in other hospitals or in nursing homes were not considered. These represent additional variables that can differentiate the precision of CCI from CDC in weighting the overall morbidity burden. Fourth, the relatively low rate of patients experiencing more than one complication may have somehow faded the difference in accuracy of the two scales. Fifth, a substantial amount of data used to calculate CCI was retrieved from patients operated before the publication of this scale. For this reason, CCI values were partially deducted by the registered complications in each center. Lastly, the local practice could have affected the approach and the decision-making strategy in dealing with a complication. Interventional or conservative treatments are graded differently in CCI and CDC and may per se affect LOS.

## 5. Conclusions

Even if both scales performed well in predicting LOS and e-LOS of patients undergoing liver resection for HCC, CCI was moderately superior to CDC. The results of this population-specific analysis suggest that CCI is preferable in reporting postoperative morbidity even though CDC metrics maintain acceptable accuracy.

## Figures and Tables

**Table 1:** Characteristics of the derivation and validation sets.

Variables	Derivation Set (*N* = 1345)		Validation Set (*N* = 1324)	
	Median [IQR] or *n* (%)	Missing (%)	Median [IQR] or *n* (%)	Missing (%)
Age	70.0 [63.0, 75.0]	1.2	69.8 [62.4, 75.1]	0.7
Females	328 (24.4)	0	271 (20.5)	0
American Society of Anesthesiologists score		2.7		4.7
1	16 (1.2)		50 (4.0)	
2	682 (52.1)		550 (43.6)	
≥3	611 (46.7)		662 (52.5)	
Cirrhosis	927 (70.5)	2.3	741 (56.1)	0.2
Child-Pugh Grade		3.9		0
A	1175 (90.9)		1294 (97.1)	
B	118 (9.1)		30 (2.3)	
Surgery duration, min	230 [170, 290]	2.3	270 [200, 370]	3.8
Blood transfusion during surgery	118 (9.1)	3.3	181 (14.5)	6.0
Laparoscopy	568 (42.2)	0	268 (21.4)	5.5
Clavien-Dindo grading		0		0
0	894 (66.5)		789 (59.6)	
I	101 (7.5)		155 (11.7)	
II	255 (19.0)		236 (17.8)	
IIIa	40 (3.0)		71 (5.4)	
IIIb	17 (1.3)		31 (2.3)	
IVa	20 (1.5)		18 (1.4)	
IVb	4 (0.3)		4 (0.3)	
V	14 (1.0)		20 (1.5)	
Comprehensive Complication Index CCI > 0	20.9 [20.9, 26.6]	0	20.9 [8.7, 29.6]	0
Length of hospitalization, days	7.0 [5.0, 9.0]	0	9.0 [7.0, 12.0]	0
HBV	276 (21.4)	4.0	213 (16.2)	0.5
HCV	714 (55.3)	4.1	544 (41.3)	0.4
Bilirubin, mg/dL	0.90 [0.63, 1.23]	1.8	0.80 [0.60, 1.01]	1.4
Albumin, g/dL	3.80 [3.50, 4.21]	3.4	4.00 [3.80, 4.30]	2.7
Platelets, 10^3/mm^3^	169.0 [117.0, 230.0]	1.8	170.0 [123.0, 223.8]	0.5
International Normalized Ratio	1.12 [1.03, 1.29]	1.9	1.08 [1.02, 1.15]	1.3
Number of nodules		0.1		3.9
1	1068 (79.5)		973 (76.4)	
2	202 (15.0)		186 (14.6)	
3	56 (4.2)		71 (5.6)	
4	12 (0.9)		25 (2.0)	
5	5 (0.4)		5 (0.4)	
≥6	1 (0.1)		13 (1.0)	
Size of bigger nodule, cm	4.0 [2.9, 6.0]	0.4	4.0 [2.5, 6.3]	1.9
Major surgery	293 (21.8)	0.3	263 (20.1)	1.1
Surgical procedure		0.5		0.2
Parenchyma-sparing resection	400 (29.9)		579 (43.8)	
Segmentecnomy	437 (32.6)		325 (24.6)	
Right hepatectomy	129 (9.6)		115 (8.7)	
Left hepatectomy	106 (7.9)		81 (6.1)	
Right posterior sectionectomy	32 (2.4)		19 (1.4)	
Right anterior sectionectomy	58 (4.3)		32 (2.4)	
Left lateral sectionectomy	85 (6.3)		52 (3.9)	
Right trisectionectomy	13 (1.0)		14 (1.1)	
Left trisectionectomy	6 (0.4)		1 (0.1)	
Other	72 (5.4)		104 (7.9)	
Number of complications		0		0
0	894 (66.5)		787 (59.4)	
1	260 (19.3)		363 (27.4)	
≥2	191 (14.2)		174 (13.1)	
Center volume		0		0
Low	108 (8.0)		113 (8.5)	
Medium	608 (45.2)		180 (13.6)	
High	629 (46.8)		1031 (77.9)	

**Table 2:** Linear regression analysis for LOS (log-transformed). The univariate models include CCI or Clavien-Dindo as the only covariate. The multivariate models are adjusted for the following covariates: type of surgery (major vs. minor), open vs. laparoscopy, age (per year), American Society of Anesthesiologists (ASA) score (1–2 vs. 3–4), Child grade (A vs. B), duration of surgery (>4 h vs. ≤4 h), center volume (high vs. medium/low). Patients died during hospital stay are excluded from all samples.

Sample	Model	Derivation Set			Validation Set	
		Mean Change (95% CI)	R^2^	RMSE	R^2^	RMSE
Overall sample Derivation set *n* = 1331. Validation set *n* = 1304.	CCI, per 10 unit increase Univariate model	0.290 (0.273;0.307)	45%	0.41	36%	0.49
	Clavien-Dindo, per category increase Univariate model	0.312 (0.292;0.332)	41%	0.43	34%	0.49
	CCI, per 10 unit increase Multivariate model	0.267 (0.251;0.282)	57%	0.37	44%	0.50
	Clavien-Dindo, per category increase Multivariate model	0.291 (0.273;0.309)	54%	0.38	43%	0.51
Subgroup of patients with at least two complications Derivation set *n* = 177. Validation set *n* = 154.	CCI, per 10 unit increase Univariate model	0.240 (0.197;0.283)	41%	0.47	15%	0.59
	Clavien-Dindo, per category increase Univariate model	0.254 (0.186;0.323)	24%	0.54	12%	0.59
	CCI, per 10 unit increase Multivariate model	0.219 (0.176;0.262)	47%	0.45	23%	0.59
	Clavien-Dindo, per category increase Multivariate model	0.223 (0.157;0.289)	32%	0.52	21%	0.63

LOS: Length of stay; CCI: Comprehensive complication index; CI: Confidence Interval; RMSE: Root mean squared error.

**Table 3:** Logistic regression analysis for e-LOS (LOS ≥ 15 days). The univariate models include CCI or Clavien-Dindo as the only covariate. The multivariate models are adjusted for the following covariates: type of surgery (major vs. minor), open vs. laparoscopy, age (per year), ASA score (1–2 vs. 3–4), Child grade (A vs. B), and duration of surgery (>4 h vs. ≤4 h), center volume (high vs. medium or low). Patients died during hospital stay are excluded from all samples.

Sample	Model	Derivation Set		Validation Set
		OR (95% CI)	AUC	AUC
Overall sample Derivation set *n* = 1331. Validation set *n* = 1304.	CCI, per 10 unit increase Univariate model	5.826 (4.402;7.709)	96.1%	87.7%
	Clavien-Dindo, per category increase Univariate model	5.298 (4.067;6.902)	94.0%	86.9%
	CCI, per 10 unit increase Multivariate model	5.590 (4.201;7.438)	96.4%	89.3%
	Clavien-Dindo, per category increase Multivariate model	5.507 (4.152;7.304)	96.2%	89.0%
Subgroup of patients with at least two complications Derivation set *n* = 177. Validation set *n* = 154.	CCI, per 10 unit increase Univariate model	3.003 (2.085;4.327)	83.0%	67.1%
	Clavien-Dindo, per category increase Univariate model	2.638 (1.794;3.878)	76.5%	65.2%
	CCI, per 10 unit increase Multivariate model	2.793 (1.896;4.115)	85.0%	67.3%
	Clavien-Dindo, per category increase Multivariate model	2.439 (1.666;3.57)	80.6%	65.8%

LOS: Length of stay; CCI: Comprehensive complication index; OR: Odds ratio; CI: Confidence interval; AUC: Area under the curve.

## Supplementary Materials

The following are available online at https://www.mdpi.com/2072-6694/12/12/3868/s1, Table S1: Multivariate linear regression models for LOS (log-transformed) on the derivation set. Table S2: Multivariate linear regression models for e-LOS (LOS ≥ 15 days) on the derivation set. Figure S1: ROC curves on subgroups of patients with at least one complication showing the performance of CCI to identify those with e-LOS. The subgroups considered are: (A) minor surgery, (B) major surgery, (C) open surgery, (D) laparoscopy surgery, (E) no cirrhosis, (F) cirrhosis, (G) Child-Pugh grade A, (H) Child-Pugh grade B. The AUC values and the optimal cut-point identified by the Youden Index with the corresponding sensitivity and specificity values are also reported.
