# Supplementary material for: Performance of Comprehensive Complication Index and Clavien-Dindo Complication Scoring System in Liver Surgery for Hepatocellular Carcinoma
Source: Cancers (Basel). 2020 Dec 21;12(12):3868. doi: 10.3390/cancers12123868 (PMC7767420; doi:10.3390/cancers12123868)
Supplement: Supplementary file 1 [file cancers-12-03868-s001.pdf]

cancers MDPI

Supplementary Material

Table S1. Multivariate linear regression models for LOS (log-transformed) on the derivation set.

Sample	Variables	CCI model	Clavien-Dindo model
		Mean change (95% CI)	Mean change (95% CI)
Overall sample n = 1331	CCI, per 10 unit increase	0.267 (0.251;0.282)	-
	Clavien-Dindo, per category increase	-	0.291 (0.273;0.309)
	Surgery major vs minor	-0.012 (-0.065;0.041)	0.006 (-0.048;0.06)
	Laparoscopy vs open	-0.142 (-0.184;-0.1)	-0.134 (-0.177;-0.091)
	Age, per year	0.0001 (-0.002;0.003)	-0.0001 (-0.003;0.002)
	ASA score 3-4 vs 1-2	0.027 (-0.018;0.071)	0.037 (-0.008;0.083)
	Child grade B vs A	0.045 (-0.028;0.118)	0.054 (-0.022;0.129)
	Duration of surgery >4h vs ≤4h	0.103 (0.058;0.147)	0.108 (0.063;0.154)
	Center volume high vs med or low	-0.315 (-0.360;-0.270)	-0.353 (-0.399;-0.308)
Subgroup of patients with at least two complications n = 177	CCI, per 10 unit increase	0.219 (0.176;0.262)	-
	Clavien-Dindo, per category increase	-	0.223 (0.157;0.289)
	Surgery major vs minor	-0.056 (-0.235;0.124)	-0.009 (-0.212;0.193)
	Laparoscopy vs open	-0.168 (-0.319;-0.018)	-0.134 (-0.305;0.036)
	Age, per year	0.001 (-0.006;0.009)	0.001 (-0.008;0.010)
	ASA score 3-4 vs 1-2	0.141 (-0.006;0.289)	0.199 (0.034;0.364)
	Child grade B vs A	0.250 (-0.039;0.539)	0.336 (-0.002;0.674)
	Duration of surgery >4h vs ≤4h	0.261 (0.115;0.408)	0.280 (0.115;0.446)
	Center volume high vs med or low	-0.220 (-0.599;0.159)	-0.241 (-0.669;0.186)

LOS: Length of stay; CCI: Comprehensive Complication Index; CI: Confidence interval.

Table S2. Multivariate linear regression models for e-LOS (LOS ≥ 15 days) on the derivation set.

Sample	Variables	CCI model	Clavien-Dindo model
		OR (95% CI)	OR (95% CI)
Overall sample n = 1331	CCI, per 10 unit increase	5.507 (4.152;7.304)	-
	Clavien-Dindo, per category increase	-	5.590 (4.201;7.438)
	Surgery major vs minor	0.810 (0.400;1.641)	1.014 (0.521;1.973)
	Laparoscopy vs open	0.623 (0.344;1.128)	0.740 (0.413;1.324)
	Age, per year	0.990 (0.961;1.021)	0.991 (0.961;1.022)
	ASA score 3-4 vs 1-2	1.712 (0.914;3.208)	1.978 (1.068;3.662)
	Child grade B vs A	0.892 (0.336;2.372)	1.336 (0.506;3.528)
	Duration of surgery >4h vs ≤4h	1.669 (0.912;3.053)	1.769 (0.989;3.162)
	Center volume high vs med or low	0.650 (0.341;1.241)	0.267 (0.133;0.533)
Subgroup of patients with at least two complications n = 177	CCI, per 10 unit increase	2.793 (1.896;4.115)	-
	Clavien-Dindo, per category increase	-	2.439 (1.666;3.57)
	Surgery major vs minor	0.779 (0.269;2.256)	1.021 (0.394;2.645)
	Laparoscopy vs open	0.551 (0.227;1.339)	0.673 (0.294;1.544)
	Age, per year	0.976 (0.937;1.016)	0.98 (0.943;1.018)
	ASA score 3-4 vs 1-2	3.093 (1.217;7.861)	3.336 (1.422;7.822)
	Child grade B vs A	2.016 (0.45;9.036)	3.469 (0.822;14.634)
	Duration of surgery >4h vs ≤4h	3.417 (1.458;8.007)	3.179 (1.449;6.975)
	Center volume high vs med or low	0.316 (0.046;2.197)	0.312 (0.046;2.124)

LOS: Length of stay; CCI: Comprehensive Complication Index; OR: Odds Ratio; CI: Confidence interval.

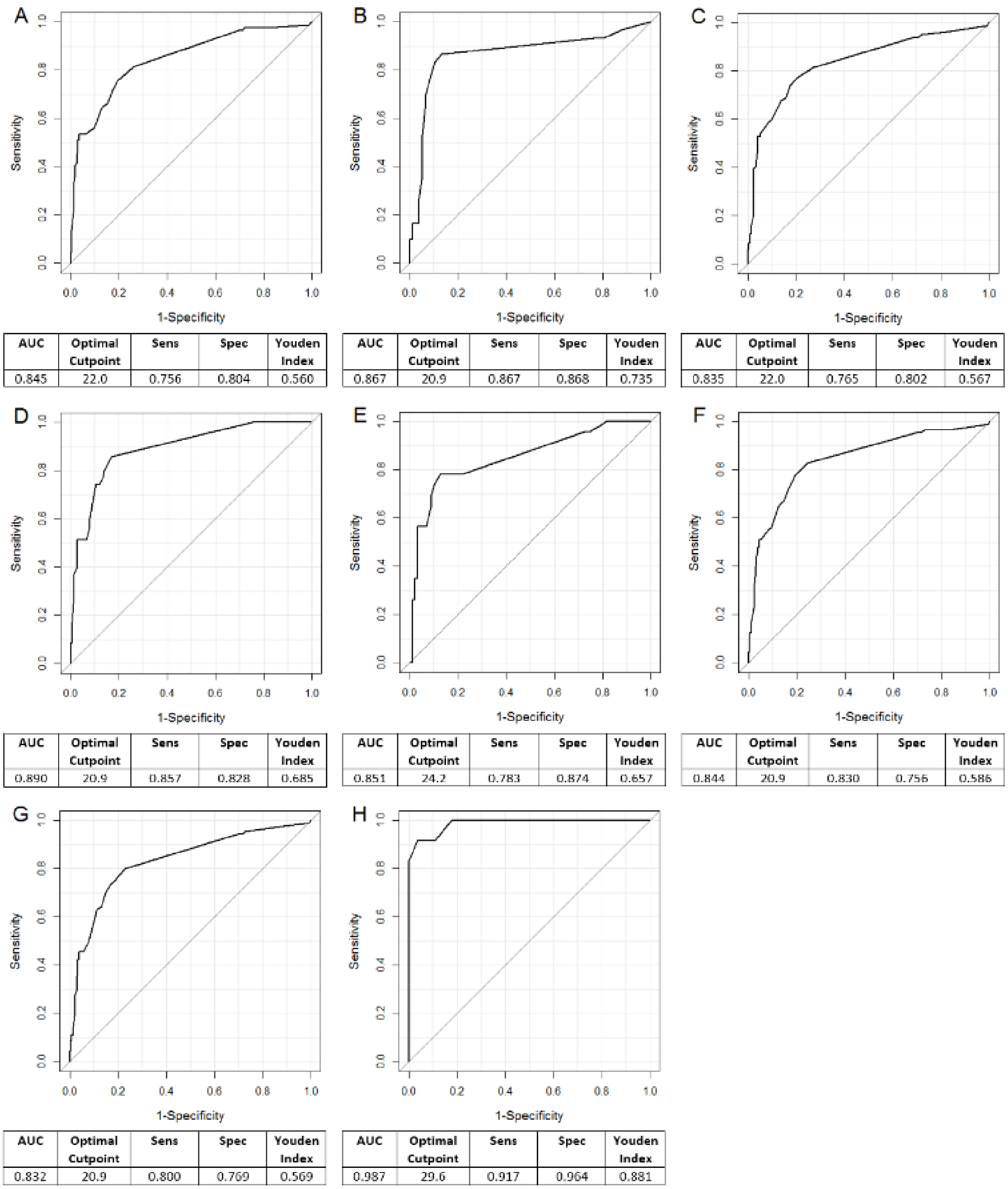

AUC	Optimal Cutpoint	Sens	Spec	Youden Index
0.845	22.0	0.756	0.804	0.560

AUC	Optimal Cutpoint	Sens	Spec	Youden Index
0.867	20.9	0.867	0.868	0.735

AUC	Optimal Cutpoint	Sens	Spec	Youden Index
0.835	22.0	0.765	0.802	0.567

AUC	Optimal Cutpoint	Sens	Spec	Youden Index
0.890	20.9	0.857	0.828	0.685

AUC	Optimal Cutpoint	Sens	Spec	Youden Index
0.851	24.2	0.783	0.874	0.657

AUC	Optimal Cutpoint	Sens	Spec	Youden Index
0.844	20.9	0.830	0.756	0.586

AUC	Optimal Cutpoint	Sens	Spec	Youden Index
0.832	20.9	0.800	0.769	0.569

AUC	Optimal Cutpoint	Sens	Spec	Youden Index
0.987	29.6	0.917	0.964	0.881

Figure S1. ROC curves on subgroups of patients with at least one complication showing the performance of CCI to identify those with e-LOS. The subgroups considered are: (**A**) minor surgery, (**B**) major surgery, (**C**) open surgery, (**D**) laparoscopy surgery, (**E**) no cirrhosis, (**F**) cirrhosis, (**G**) Child-Pugh grade A, (**H**) Child-Pugh grade B. The AUC values and the optimal cut-point identified by the Youden Index with the corresponding sensitivity, specificity, positive and negative predictive values are also reported.
